# Electronic and Optical Properties of InAs QDs Grown by MBE on InGaAs Metamorphic Buffer

**DOI:** 10.3390/ma15031071

**Published:** 2022-01-29

**Authors:** Paweł Wyborski, Paweł Podemski, Piotr Andrzej Wroński, Fauzia Jabeen, Sven Höfling, Grzegorz Sęk

**Affiliations:** 1 Department of Experimental Physics, Faculty of Fundamental Problems of Technology, Wrocław University of Science and Technology, Wybrzeże Wyspiańskiego 27, 50-370 Wrocław, Poland; pawel.podemski@pwr.edu.pl (P.P.); grzegorz.sek@pwr.edu.pl (G.S.); 2 Technische Physik, Wilhelm-Conrad-Röntgen-Research Center for Complex Material Systems, University of Würzburg, Am Hubland, D-97074 Würzburg, Germany; piotr-andrzej.wronski@physik.uni-wuerzburg.de (P.A.W.); fauzia.jabeen@physik.uni-wuerzburg.de (F.J.); sven.hoefling@physik.uni-wuerzburg.de (S.H.); 3 Faculty of Engineering and Physical Sciences, University of Southampton, Southampton SO17 1BJ, UK

**Keywords:** molecular beam epitaxy, quantum dot, metamorphic buffer layer, band structure, photoluminescence, photoreflectance

## Abstract

We present the optical characterization of GaAs-based InAs quantum dots (QDs) grown by molecular beam epitaxy on a digitally alloyed InGaAs metamorphic buffer layer (MBL) with gradual composition ensuring a redshift of the QD emission up to the second telecom window. Based on the photoluminescence (PL) measurements and numerical calculations, we analyzed the factors influencing the energies of optical transitions in QDs, among which the QD height seems to be dominating. In addition, polarization anisotropy of the QD emission was observed, which is a fingerprint of significant valence states mixing enhanced by the QD confinement potential asymmetry, driven by the decreased strain with increasing In content in the MBL. The barrier-related transitions were probed by photoreflectance, which combined with photoluminescence data and the PL temperature dependence, allowed for the determination of the carrier activation energies and the main channels of carrier loss, identified as the carrier escape to the MBL barrier. Eventually, the zero-dimensional character of the emission was confirmed by detecting the photoluminescence from single QDs with identified features of the confined neutral exciton and biexciton complexes via the excitation power and polarization dependences.

## 1. Introduction

Semiconductor self-assembled epitaxial quantum dots (QDs) have been demonstrated to be suitable candidates for use in a wide range of optoelectronic devices, from lasers [1,2], optical amplifiers [1,3], or other broadband sources [4,5] to quantum computing and quantum information processing employing QD-based non-classical emitters [6,7,8,9,10]. In particular, the possibility of controlling the parameters of QDs via the materials choice, and the related band structure and strain engineering, makes them attractive for applications due to the possibility of obtaining compatibility with the existing silica-based optical fiber infrastructure and emission in the high-transmission spectral range of telecommunication windows, characterized by the lowest attenuation in the third telecom or minimum optical signal dispersion in the second telecom windows [10,11].

The emission in the telecom range is typically obtained for QDs made of InAs material on an InP substrate. There exist many demonstrations concerning both the laser structures [1] and the non-classical quantum emitters [12,13,14,15,16,17,18]. However, the InP-based technology has its drawbacks—it is still relatively expensive and, in some aspects, less developed than the very mature GaAs-based technology. For instance, fabrication of efficient photonic structures, including distributed Bragg reflectors (DBRs) adapted to the InP material is significantly limited due to the lack of substrate-lattice-matched materials with large enough refractive index contrast, assuring simultaneously good structural and optical quality. On the other hand, there are well developed solutions based on the GaAs material system [8]. However, the difference between lattice constants of the InAs (QD layer) and GaAs (substrate) is relatively high, resulting in the technologically demanding growth of GaAs-based QDs emitting in the telecom range—the standard InAs/GaAs QDs emit in the range below 1.1 µm (850–1000 nm) [6,9,10]. To extend the spectral range of emission, it is necessary to apply additional steps to modify the properties of the dots, mostly employing the strain engineering or modification of the QDs’ size or composition [11]. There exist at least several approaches allowing for the shifting of the emission wavelength to the telecommunication windows, for instance by using vertically-stacked QDs [19,20,21,22,23], growth up to the second critical thickness [24,25], controlled overgrowth of InGaAs QDs [26], adding small concentrations of nitrogen to the InAs QDs [27], applying the strain-reducing layer [28,29,30,31,32,33,34,35,36,37,38,39,40,41,42], or the metamorphic-buffer-layer (MBL) [5,11,43,44,45,46]. Most of these solutions concern the use of QDs as an active region in lasers or amplifiers, while the practical demonstrations employing single GaAs-based QDs as an active element of non-classical emitters for quantum technology applications in the telecom range are still under development. Furthermore, the only approach reported as suitable to obtain single-photon emission from InAs dots on the GaAs substrate in both the 2nd and 3rd telecommunication windows is the use of MBL and the related strain engineering [11,44,45,46]. Previous results concerned mainly MBL-based QD structures grown by metal-organic vapor-phase epitaxy (MOVPE) [45], which allowed for the demonstrating of important quantum communication milestones, such as the emission of indistinguishable photons [47,48]—also in the on-demand mode [49], generation of pairs of polarization-entangled photons [50], or the possibility of precise piezo-tuning [51]. For QDs grown by the molecular beam epitaxy (MBE), single-photon emission has been shown only in the third telecom window so far [46]. On the other hand, there are reports presenting the suitability of the MBL approach in the MBE growth of GaAs-based ensembles of QDs for laser applications in the telecom spectral range [43,52].

Here, we present a systematic optical characterization of InAs QDs grown by MBE on a GaAs substrate and on an InGaAs MBL with the compositional gradient. The work concerns the influence of the MBL structure on the dots’ optical properties and the energy structure of the entire system. Based on the experimental results, complemented by the numerical band structure calculations, the possibility of controlling the emission wavelength by tailoring the indium content and the gradient within the MBL is demonstrated. The influence of the MBL composition on the observed linear polarization of emission, indicating the existence of asymmetry of the confinement potential, is also observed. Additionally, based on the spectral features detected in the absorption-like (photoreflectance) and emission-like (photoluminescence) spectra, the major optical transitions could be determined and then combined with the results of the PL temperature dependence analysis in order to identify the main carrier loss mechanisms. Eventually, high-spatial-resolution photoluminescence measurements allowed the observation of emission from the neutral exciton and biexciton complexes confined in single quantum dots of that kind.

## 2. Materials and Methods

**Figure 1:**
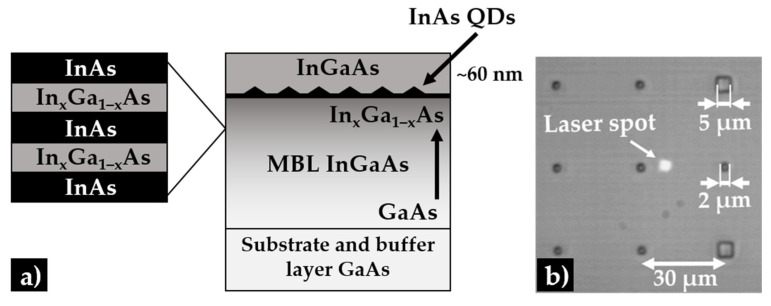
(**a**) Scheme of the investigated structure with InAs/InGaAs/GaAs QDs; (**b**) optical microscope image of mesa structures with a matrix of 2 μm and 5 μm (reference) mesas and a laser spot visible on the sample surface.

The investigated structures were grown by MBE on a GaAs (001) substrate. In all samples the sequence of epitaxial layers shown in Figure 1a begins with 400 nm of the GaAs buffer layer.

Next, a linearly-graded In_x_Ga_1−x_As MBL with maximal In content in the top part of the layer was grown (see Table 1). The composition of the MBL was controlled via the digital alloy approach. It began with 30 nm layer of InGaAs made of 75 repetitions of 0.4-nm-thick GaAs layers with 0.05 Å InAs insertions to get approx. 1% of In content. Next, the width of insertions was modified whereas the GaAs width was kept constant, allowing for a gradual increase in the In content. It was continued until the maximum value of the In content in the upper part of the MBL layer was reached. The graded InGaAs alloy acts as a substrate to grow optically-active InAs QDs red-shifted to the telecom range [46]. On top of the MBL, 1.5 monolayers of InAs material were deposited to form QDs. The dots were covered with a 60-nm-thick InGaAs layer, with slightly lower indium content than in the upmost part of the MBL. In order to make the characterization of single QDs possible, repeatable and to limit the number of probed QDs in a single-dot optical experiment, a combination of electron beam lithography and wet etching was used to fabricate mesa structures of a cylindrical shape, with various diameters ranging from 200 to 3000 nm and with a height of about 100 nm. The distance between mesas is 30 μm, as designed in the lithography mask. It can be also seen in Figure 1b, where an example of image from the experimental setup optical microscope is shown. The mesa spacing is significantly larger than the laser spot diameter, so it is possible to observe an emission from a single mesa at a time.

Optical characterization of planar (unpatterned) structures with photoluminescence (PL) and photoreflectance (PR) was performed using standard experimental setups [53] and by employing 532 nm line of a continuous-wave (CW), frequency-doubled neodymium-doped yttrium aluminum garnet laser (CNI Laser, Changchun, China, MGL-III-532–200 mW) as a non-resonant excitation/modulation source. A 0.3-m-focal-length spectrometer (Teledyne Princeton Instruments, Acton, MA, USA, Acton SP2300i) equipped with a liquid-nitrogen-cooled InGaAs 1024 pixels linear array detector (Teledyne Princeton Instruments, Acton, MA, USA Acton OMA V:InGaAs) was used for PL measurements and thermoelectrically-cooled long-wavelength InGaAs photodiode (Hamamatsu, Hamamatsu City, Japan, C12483-250) combined with a lock-in amplifier (Stanford Research Systems, Sunnyvale, CA, USA, SR830) were employed as the detection system in PR measurements.

In order to characterize single QDs, samples with additionally formed mesas were used. They were probed by high-spatial-resolution photoluminescence (µPL) in a liquid-helium continuous-flow cryostat (Lake Shore Cryotronics, Westerville, OH, USA, JANIS ST-500) at the temperature of 5 K, minimizing the thermally-activated carrier losses and the influence of acoustic phonons on the spectral lines broadening. Non-resonant excitation was provided by a CW 660 nm semiconductor laser (Coherent, Santa Clara, CA, USA, CUBE) focused on the sample surface by a microscope objective (Mitutoyo, Aurora, CO, USA, M Plan Apo NIR 20x, NA = 0.4, infinity corrected, 20 mm working distance) to a beam diameter of the order of single micrometers allowing for the illumination of one mesa at a time and obtaining the spatial resolution below 2 μm (defined by the diffraction-limited laser spot size). On the detection side, the setup was equipped with a 1-m-focal-length spectrometer (HORIBA Scientific, Kyoto, Japan, FHR 1000) coupled to a liquid-nitrogen-cooled InGaAs 1024 pixels linear array detector (HORIBA Scientific, Kyoto, Japan, Symphony II), providing the spectral resolution below 25 µeV, which is sufficient for resolving single-dot emission lines.

## 3. Results and Discussion

**Figure 2:**
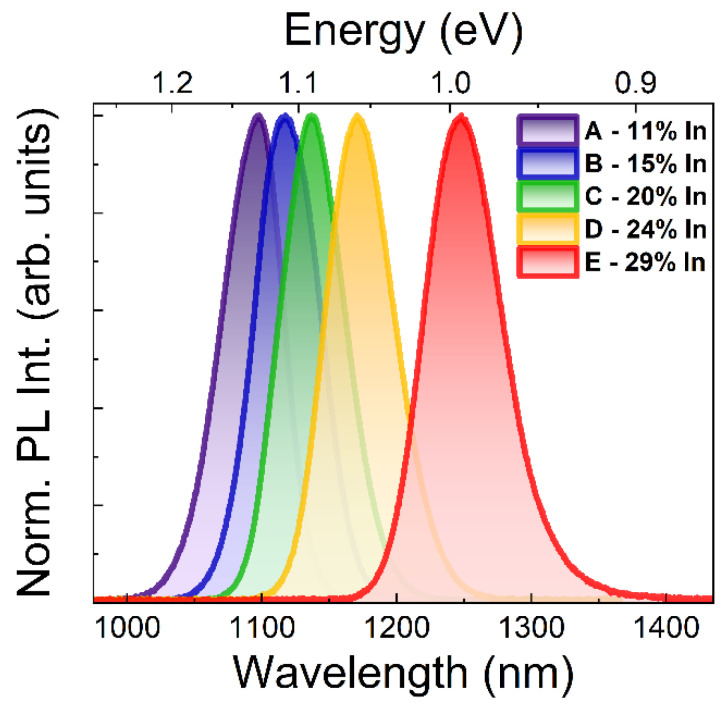
Normalized PL spectra for structures A–E (temperature: 10 K, excitation power: 100 μW, excitation wavelength: 532 nm, CW).

Figure 2 presents low-temperature PL spectra for five structures (A–E) with increasing indium content in the top part of the MBL layer, which show the emission from QDs at the wavelengths from below 1.1 µm for a sample A with 11% In content, to almost 1.3 µm for the structure with the highest indium content (sample E: 29% In).

The approach with a gradient of the composition in the MBL layer allows for the obtaining of a good quality material—the grown QDs are characterized by a bright emission, which maintains similar PL signal intensity even for structures with the highest indium content (the intensity differences between the samples does not exceed 30%). The width of the PL peaks is approx. 55 meV in all cases, which suggests the similar structural quality of the QD material for all samples. It is most probably dominated by fluctuations in the QD heights being the strongest confinement direction. However, depending on the value of the height it can correspond to a different range of heights distribution (see the discussion below).

The main mechanism driving the spectral shift of the QD emission is related to the reduction of the difference of lattice constants of the buffer material preceding the QD layer. This is about 5% between InAs and In_0.29_Ga_0.71_As or even less if the QD material is not pure InAs (about 3% if the dots’ composition is In_0.7_Ga_0.3_As), when compared to 7% for InAs and GaAs. It means it becomes closer to the value typical for the InAs on InP substrate (approx. 3% of the lattice constants difference). Lower mismatch between lattice constants of the QD material and the layer underneath typically results in growth of larger self-assembled dots [54,55], which translates into QD emission redshift. Additionally, in MBE growth, a tendency to increased nanostructures’ lateral shape anisotropy is very often observed when the strain is lower [54]. This is also accompanied by other effects influencing the QD emission energy. Tailoring the indium content in MBL changes the height of the barrier and affects the QD confined levels via changed contribution of the strain to the confinement potential.

**Figure 3:**
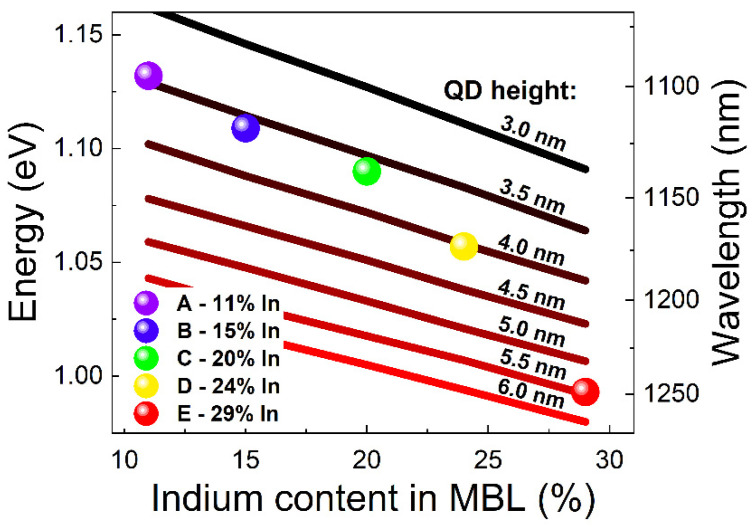
Comparison of the emission energy from PL measurements for structures A–E (dots) with the results of numerical simulations for QD height from 3 to 6 nm with 0.5 nm step (lines).

To determine the influence of the above-mentioned factors on the emission energy, we performed numerical electronic structure calculations employing the 8-band ***k***·***p*** method [56] for the symmetrical, lens-shaped QDs on a 0.5-nm-thick wetting layer. Due to the lack of respective data on the exact QD compositions, we assume there is similar In content in all samples. However, the commonly occurring materials’ intermixing means that the QDs are never made of pure InAs. Therefore, we assumed an average indium content of 70% for these calculations, following the literature data [57]. Figure 3 compares the experimental emission energies of the investigated QDs with energies from simulations for different indium content in the MBL versus the QD height. Changing the height corresponds to modifying the base size of the truncated sphere proportionally, while keeping the aspect ratio constant (on the level of 5 in our case). However, the effect of the height, as the strongest confinement direction, on the energy of confined levels is predominant. These calculations show the general trends to indicate the probable mechanism of spectral shift observed in the analyzed structures.

The presented comparison confirms the importance of the modifications in the MBL properties connected with two processes related to the shift in emission energy: the modification of the QD size and the modification of the indium content in the MBL layer (change of strain and energy bandgap of InGaAs being a confinement barrier for the dots). We observe that for a fixed QD height, changes of the MBL composition from 11% to 29% allows for the reduction of the QDs emission energy by about 50 meV at most, while the modification of the QD height in the range from 3 to 6 nm (with 0.5 nm step) causes a transition energy reduction up to 200 meV. This suggests a greater impact of the modification of QD size, especially in the range above 20% of the In content in the MBL.

Comparing the experimentally obtained widths of the PL peaks (approx. 55 meV for all samples) with the simulations on the energy vs. height dependence, we can notice that the energy distribution for the highest transition energies translates to a height distribution of about 3.5 ± 0.5 nm, which means a percentage of fluctuations at the level of ±15%, while at the low energy side of this dependence corresponds to the height distribution of about 5.5 ± 1.0 nm, which translates into fluctuations of around ±18%. This indicates a similar level of the relative size distribution for all of the investigated structures.

**Figure 4:**
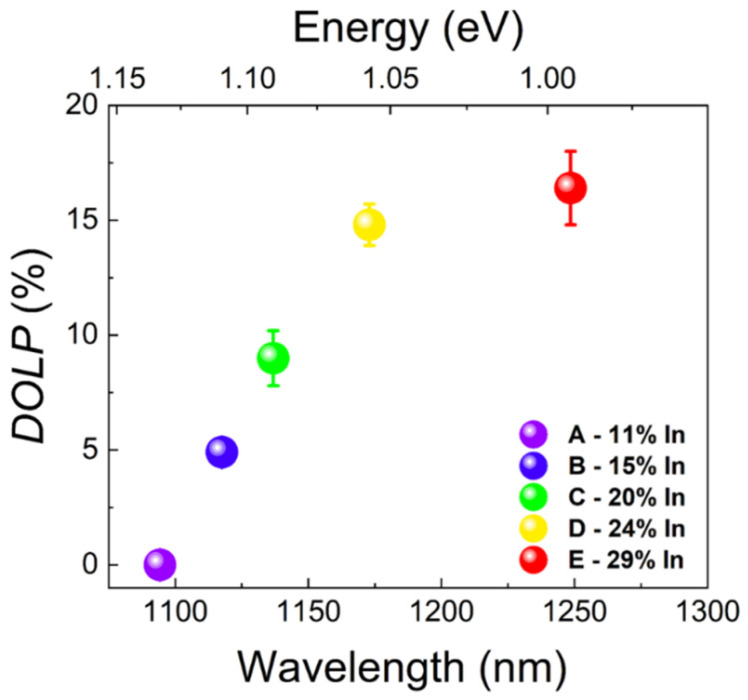
Degree of linear polarization of emission from QDs as a function of emission wavelength with error bars representing the standard error from the fit with Equation (1).

In order to find out about the possible polarization anisotropy of emissions from the investigated QDs, we measured the dependence of the PL signal on the linear polarization (for full experimental data see the Supplementary Materials: Figure S1). The obtained dependence was fitted with the following Formula [58]:

(1) I(θ)=A(1+DOLP·cos(2(θ−ϕ)))

where I(θ) is the intensity of PL as a function of the polarization angle θ, *A* is the scaling factor, ϕ is an offset from the 0 angle and *DOLP* is the degree of linear polarization of emission from QDs, defined as $DOLP=\left(I_{max}-I_{min}\right)/\left(I_{max}+I_{min}\right)$. Figure 4 shows the experimentally obtained *DOLP* value from the polarization-resolved measurements for all the examined structures.

It can be seen that an increase in the indium content in the MBL induces not only the spectral shift of the QD emission but also leads to the increase in the value of the degree of linear polarization from a close to 0% value for the structure with the lowest In content in the MBL (emission at the energy of 1.133 eV (1095 nm)), most likely corresponding to almost fully symmetric QDs, to the *DOLP* value of 16.5% for the structure with PL peak at the energy of 0.993 eV (1250 nm)—the largest In content in the MBL. Changes in the strain conditions during the growth of QDs from sample to sample while increasing the In content in the buffer layer underneath may lead to inducing an in-plane asymmetry of QDs’ shape or strain anisotropy, both affecting the mixing of valence band states (where the light and heavy-hole states mixing is of crucial importance), leading to a non-zero *DOLP* of QD emission [59]. The possible QD shape asymmetry is characteristic for the QDs’ growth under lower strain, and hence the stronger influence of the atomic steps on growth anisotropy (along the crystallographic directions [110] and [1,2,3,4,5,6,7,8,9,10]) may occur, as it was already observed for other types of dots grown by MBE in conditions of smaller difference in the lattice constants between the substrate/buffer and the QD materials [54,55]. It is difficult to resolve which of the parameters (QD shape asymmetry or strain anisotropy) is the main factor responsible for the observed *DOLP* change, especially for inhomogeneous systems where the differences in dot-to-dot shape and strain variations can be significant and only average properties can be specified for an ensemble of QDs [59,60,61], but one can expect that both do contribute.

**Figure 5:**
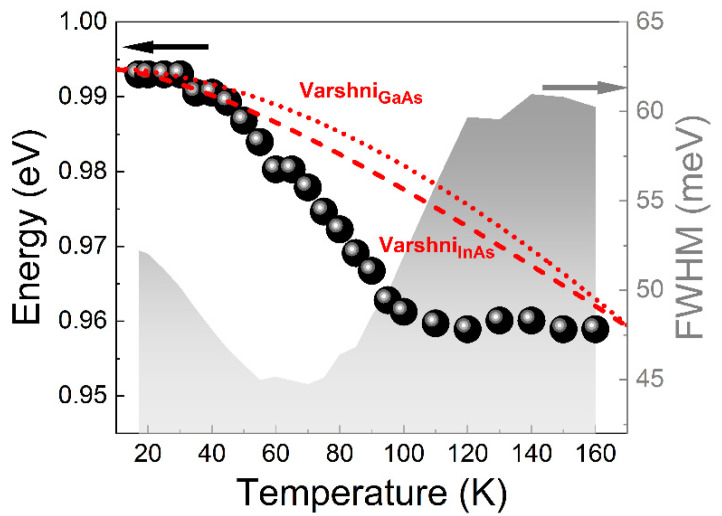
Emission energy (left axis, black dots) of QDs in structure E with Varshni dependence for GaAs (red, dotted line) and InAs (red, dashed line) with energy shifted to match the low-temperature QD emission, and full width at half maximum (FWHM) of the QD emission (right axis, gray fill) with respect to the temperature.

To further analyze the electronic structure and to identify the carrier loss mechanisms in these dots, the temperature dependence of photoluminescence was measured (for full experimental data see Supplementary Materials: Figure S2). Figure 5 shows exemplary results with the emission peak energy and the full width at half maximum (FWHM) of the PL vs. temperature for the ensemble of QDs for the sample E (with the largest maximal indium content in the MBL).

**Figure 6:**
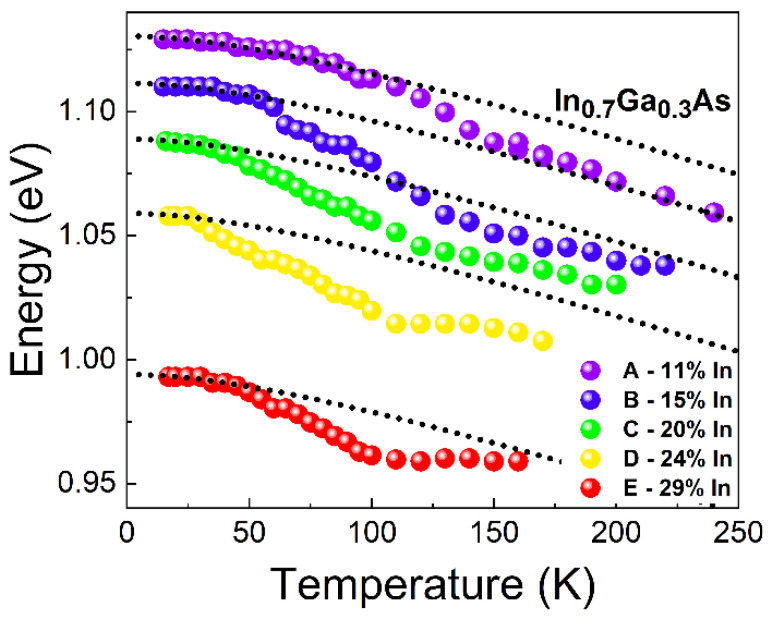
Emission energy for A-E structures (colored dots) in a function of the temperature, compared with the Varshni dependences for In_0.7_Ga_0.3_As QDs (dotted line) shifted to the corresponding QD emission energies at the lowest temperature (A: 1.132 eV, B: 1.109 eV, C: 1.09 eV, D: 1.057 eV, E: 0.993 eV).

The dependence of the emission energy on temperature is mainly related to the change of the energy gap of the QD material, which for bulk materials is described by the Varshni relation:

(2) $$E_{g}=E_{0}-\frac{\alpha T^{2}}{T+\beta }$$

where *E*_0_ is the energy bandgap of the material at the temperature of 0 K, *T* is the temperature, *α* and *β* are material parameters ($\alpha =8.871\cdot 10^{-4}\;\mathrm{eV}/K,\;\beta =572\;K$ or $\alpha =3.158\cdot 10^{-4}\;\mathrm{eV}/K,\;\beta =93\;K$ for GaAs or InAs, respectively [62]). In Figure 5 we observe a clear deviation of the PL peak shift from the reference Varshni dependences (for both: GaAs and InAs). Simultaneously, we observe a non-standard dependence of FWHM in the function of temperature: decrease of the PL peak width for temperatures in the range of 17–70 K, and then an increase above 70 K which would typically be expected in the entire range of temperatures. Both of those results are characteristic for the redistribution of carriers between QDs of the entire ensemble [63]. The thermal activation of carriers causes their redistribution to the dots with lower ground state energy (these are larger dots typically), which gives the additional redshift factor making the PL peak energy decrease faster than the one described by Varshni expression for *E*_g_. Usually it is also a reason for decreasing FWHM—after redistribution of carriers only a sub-ensemble of all QDs (larger dots with narrower size distribution) is emitting. This behavior has already been observed for different kinds of self-assembled QDs [63,64,65]. However, it is only present if the excitation power density is not too high to fill up higher QD states, which efficiently prevents the redistribution process. For our case, this effect was observed for all of the investigated structures, as shown in Figure 6.

It can be seen that the redistribution process begins at lower temperatures for structures with larger indium content, which may be related to the reduction of the activation energy for the thermally-induced transfer of carriers, most probably due to the lower energy difference between the QD ground state and the band edges of the InGaAs barrier (top of the MBL layer).

The analysis of the emission intensity from a QD ensemble as a function of temperature allowed us to determine the activation energies of carriers confined in QDs. Here, they correspond to a process of carriers’ escape from QDs, so they do not contribute to QD-related PL anymore, which is in contrast to the redistribution processes described above where the carriers are recaptured by other dots and hence contribute to the total observed PL intensity via radiative recombination in a different dot but still within the ensemble.

The obtained PL intensity temperature dependences were fitted with the Arrhenius Formula for a single activation process [66]:

(3) $$I=\frac{I_{0}}{1+A\;\exp\left(-\frac{E}{k_{B}T}\right)}$$

where, $I_{0}$ is the emission intensity at *T* = 0 K, *T* is the temperature, *E* is the activation energy, *A* is a scaling factor and $k_{B}$ is the Boltzmann constant. The resulting activation energy values are summarized in Table 2.

We obtained a clear reduction of the activation energy with the decrease in the QDs emission energy (i.e., in line with the increase of the indium content in the top part of the MBL structure).

**Figure 7:**
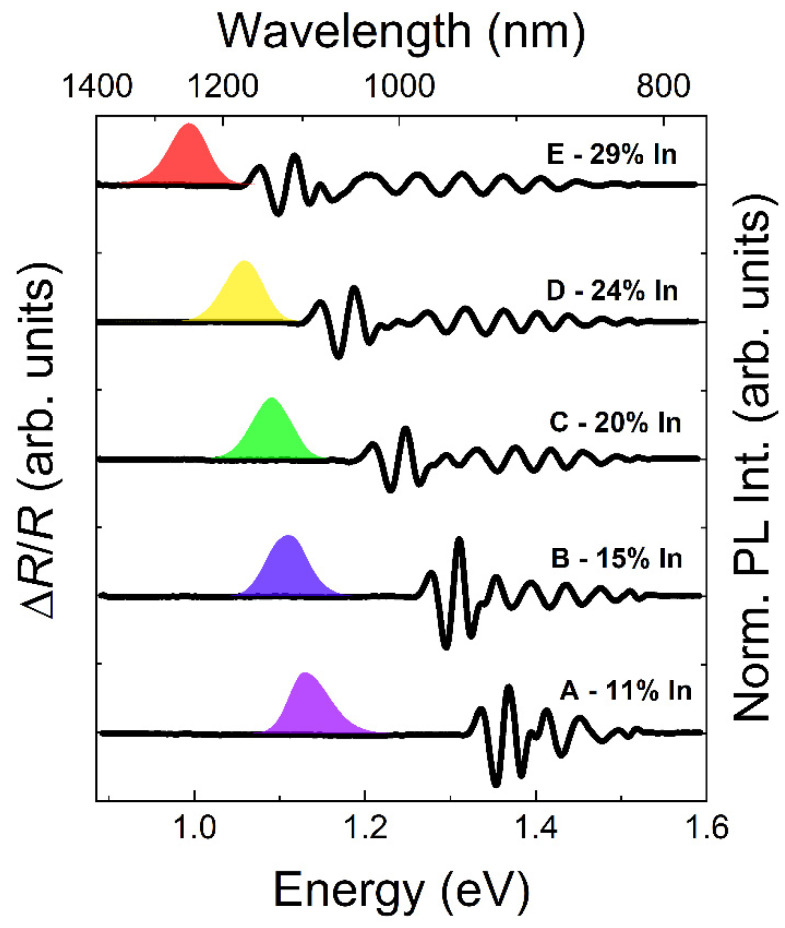
Comparison of room-temperature photoreflectance spectra shifted spectrally to low temperature (left axis) and normalized low temperature PL spectra (right axis) for A–E structures.

PR measurements (an absorption-like method) allowed for the determining of the optical transitions, which have significant intensity (oscillator strength). This technique primarily probes the transitions in bulk-like layers, which here allowed for the detection of the MBL structure bandgap (in its top part at least). Figure 7 shows PL and PR spectra for all of the samples. The presented PR spectra were measured at room temperature (T ~300 K) to avoid the influence of the efficient emission signal from QDs (which is a spurious background in PR measurements) and then shifted to higher energies according to the Varshni formula for the given InGaAs composition, in order to directly compare the PR spectra with low-temperature PL. This analysis could not be made based on PR or PL spectra exclusively, because we do not observe PL from MBL (most likely due to the efficient processes of capturing carriers from the MBL by lower energy states in the dots as well as partly by the reabsorption of the possible MBL emission by the QD layer). On the other hand, it is always challenging to receive a QD response in PR spectra due to the low QDs’ absorption (low total volume of the QD material) combined with the large spectral broadening induced by ensemble inhomogeneity. This becomes even more challenging at low temperatures when strong PL from the dots overlaps with the PR signal.

**Figure 8:**
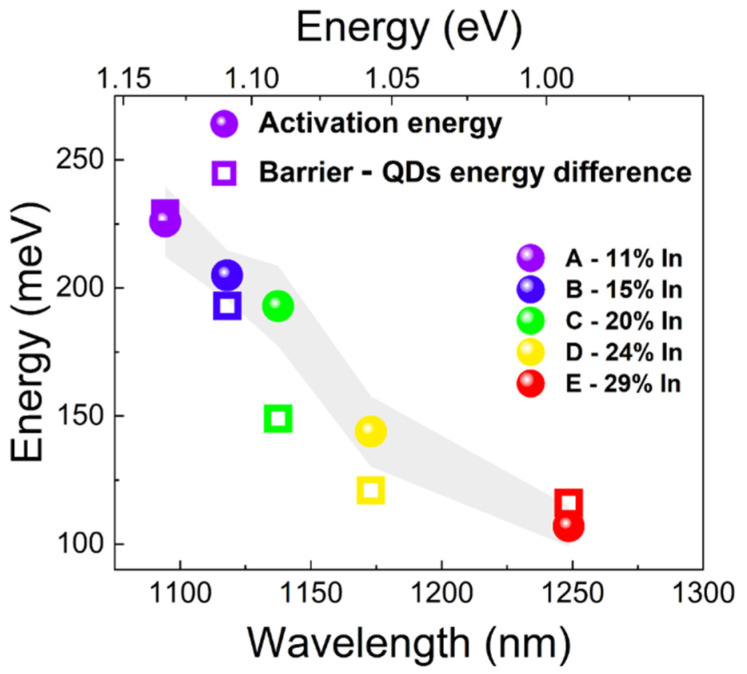
Comparison of the activation energies and the energy difference between the InGaAs barrier layer and QD ground states as a function of the QD emission wavelength. The gray shade represents the 95% confidence interval based on the standard error from the fit using Equation (3).

For all the structures, we observe a clear resonance at the low-energy end of the PR spectra. It is related to the absorption edge of the InGaAs barrier layer (top part of the MBL with the highest indium content). It cannot be a response from the cap layer which has lower indium content (larger bandgap). Resonances seen at higher energies come from the MBL (layer with the composition gradient), as well as from the cap layer. All these signals are expected to give a Franz-Keldysh-like response in the modulated reflectivity spectra [67,68,69], and will overlap each other. However, on the basis of the obtained PR spectra, we could determine the energy gap of the InGaAs barrier layer (top part of the MBL) for all the structures by using the simple three-point method [70]. Considering the energetic position of the emission from QDs, the values of the energy difference between the InGaAs barrier bandgap energy and the QD ground state transition could be derived—they are summarized together with the QD activation energy values in Figure 8.

The activation energies received from the temperature-dependent PL and the energy differences between the InGaAs barrier layer and QD ground states show a reasonable agreement. It explains the quenching process—with the temperature increase the carriers start to efficiently depopulate the ground states of QDs reaching the lowest energy levels in the barrier. Additionally, it is clearly observed that with an increase in the indium content in the MBL, the barrier-QD energy difference decreases (from 229 to 116 meV for structures A and E, respectively). It translates into effectively shallower carrier confinement potential in the longer-wavelength-emitting QDs, which is responsible for the reduction of the PL intensity from QDs at higher temperatures. It is an important issue which has to be considered in the design of optoelectronic devices for higher-temperature operation.

**Figure 9:**
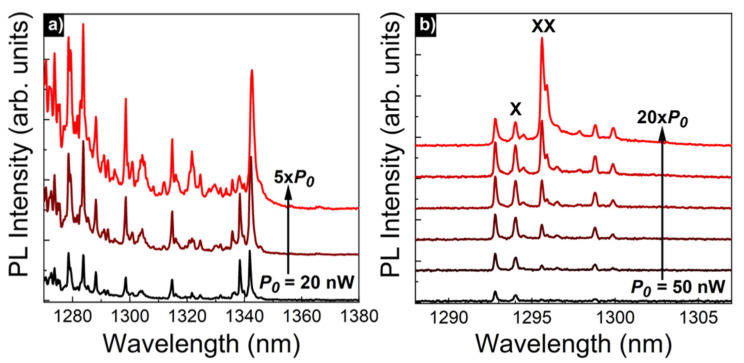
(**a**) µPL spectra in the range of 2nd telecom window from a planar structure for non-resonant excitation with power from 20 to 100 nW; (**b**) µPL spectra from QDs in mesa structure (diameter ~1 µm) for non-resonant excitation with power range 50–1000 nW (sample E).

To prove that the emission comes from the radiative recombination in single QDs and thus to confirm its zero-dimensional character, additional PL measurements were carried out using a µPL experimental setup providing high-spatial-resolution. Figure 9a shows an example of spectra from an ensemble of QDs for several excitation powers: the observation of sharp emission lines confirms the successful growth of zero-dimensional nanostructures emitting in the second telecom window (sample E).

**Figure 10:**
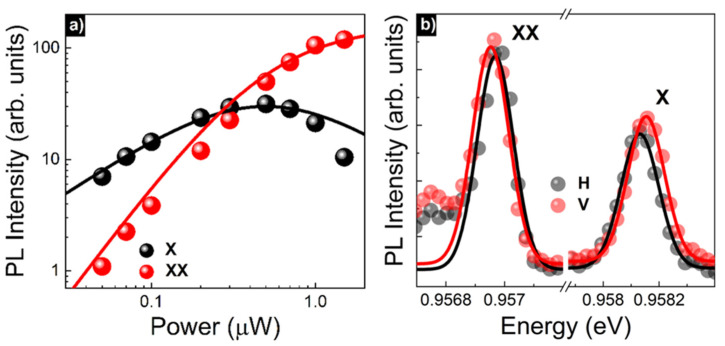
(**a**) Intensity of the exciton (X) and biexciton (XX) lines from Figure 9b in a function of excitation power with a three-level rate-equation model fit (solid line). (**b**) µPL spectra of X and XX lines from Figure 9b for two perpendicular linear polarizations (H and V).

High number of the observed emission lines is related to the relatively high surface density of QDs (~10^10^ cm^−2^) [46], so without surface patterning the observation of single QDs is possible mainly in the tails of the entire emission band (using the advantage of the QD size distribution). Figure 9b shows the excitation-power dependence (for full experimental data see Supplementary Materials: Figure S3) of the emission from single dots in a patterned structure (mesa diameter ~1 µm) in the spectral range of the second telecom window. Two of the lines were marked as corresponding to the recombination of neutral exciton (X) and biexciton (XX) complexes in a single QD, based on the analysis of the emission intensity dependence on the excitation power and polarization-resolved spectra (both presented in Figure 10). The remaining lines may be related to the emission of other excitonic complexes from the same QD or emission from other QDs in this mesa, which is possible due to the self-assembled nature of QDs, resulting in QD size distribution within the ensemble. The exact origin of all the lines could be further investigated, e.g., by measurements of the second-order correlation function (photon emission cross-correlation for different lines); however, it is an demanding experiment and the identification of all the complexes is beyond the scope of this work.

The origin of the X and XX lines was identified based on matching the dependence of the emission intensities on the excitation power to a three-level rate-equation model (Figure 10a) [71], as well as from the anti-phase dependence of the energy position of these lines in a function of the linear polarization (Figure 10b). Both of these confirm the correlation characteristic for a pair of exciton and biexciton lines from the same dot [72]. Additionally, from the energetic separation of X and XX lines, the biexciton binding energy of ~1.2 meV was determined, which is comparable to the values observed for standard InAs/GaAs QDs [73].

## 4. Conclusions

We have investigated the optical properties of structures with InAs QDs on a graded MBL with a different indium content. The structures were grown by MBE on a GaAs substrate by using a digital alloying approach. Spectroscopic measurements supported by the results of semi-quantitative numerical simulations showed the possibility of shifting the QDs energy emission, driven mainly by the modification of the QDs size, where the use of 29% indium allowed the shifting of the QDs energy to the range of the second telecommunication window. The influence of the MBL structure on the polarization properties of QD emission was observed and related to the changes in the QD shape and strain anisotropy with increasing indium content in the MBL. Based on the determined MBL ground state energy and the temperature dependences of the emission intensity, an efficient process of the carriers’ escape to the barrier states was demonstrated, with the process of redistribution of carriers within the ensemble also present. The performed µPL measurements allowed for demonstration of the emission from single QDs at 1.3 μm, confirming the zero-dimensional character of the investigated structures. PL lines related to exciton and biexciton from the same dot could be recognized, and hence the biexciton binding energy was determined. The obtained results show the potential of such GaAs-based QDs grown by MBE on an MBL as emitters in the telecom spectral range. We hope the study will stimulate further optimization work of this kind of QD material towards real-life applications, including those requiring the practical non-classical light sources for the quantum communication schemes in fiber networks.

## Figures and Tables

**Table 1:** Structure parameters: indium content and the thickness of MBL layer.

Sample	In Content in Top of the MBL: x	MBL Thickness (nm):
A	11	600
B	15	780
C	20	960
D	24	1140
E	29	1140

**Table 2:** Activation energies of the PL quenching process.

Sample	Activation Energy (meV)
A—11% In	226
B—15% In	205
C—20% In	193
D—24% In	144
E—29% In	107

## Data Availability

The data presented in this study are available on a reasonable request from the corresponding author.

## Supplementary Materials

The following supporting information can be downloaded at: https://www.mdpi.com/article/10.3390/ma15031071/s1, Figure S1: PL spectra for structures A–E in the function of linear polarization angle; Figure S2: PL spectra for structures A–E in a function of temperature; Figure S3: μPL spectra for a selected mesa in a function of the excitation power.
