# Supplementary material for: Electronic and Optical Properties of InAs QDs Grown by MBE on InGaAs Metamorphic Buffer
Source: Materials (Basel). 2022 Jan 29;15(3):1071. doi: 10.3390/ma15031071 (PMC8839711; doi:10.3390/ma15031071)
Supplement: Supplementary file 1 [file materials-15-01071-s001.zip › materials-1496174-SI.pdf]

materials

MDPI

*Article*

# Electronic and Optical Properties of InAs QDs Grown by MBE on InGaAs Metamorphic Buffer

**Paweł Wyborski [1,*], Paweł Podemski [1], Piotr Andrzej Wroński [2], Fauzia Jabeen [2,3], Sven Höfling [2] and Grzegorz Sęk [1]**

[1] Department of Experimental Physics, Faculty of Fundamental Problems of Technology, Wrocław University of Science and Technology, Wybrzeże Wyspiańskiego 27, 50-370 Wrocław, Poland; pawel.podemski@pwr.edu.pl (P.P.); grzegorz.sek@pwr.edu.pl (G.S.)
[2] Technische Physik, Wilhelm-Conrad-Röntgen-Research Center for Complex Material Systems, University of Würzburg, Am Hubland, D-97074 Würzburg, Germany; piotr-andrzej.wronski@physik.uni-wuerzburg.de (P.A.W.); fauzia.jabeen@physik.uni-wuerzburg.de (F.J.); sven.hoefling@physik.uni-wuerzburg.de (S.H.)
[3] Faculty of Engineering and Physical Sciences, University of Southampton, Southampton SO17 1BJ, UK
* Correspondence: pawel.wyborski@pwr.edu.pl

## Introduction

Here, we present the results of photoluminescence (PL) measurements of all structures A-E (see Table 1), on the basis of which the temperature dependence of PL (Figure 5, 6 and Table 2) and linear polarization dependence of PL (Figure 4), presented in the main text of the manuscript, were determined. Additionally, we attach full experimental data of microphotoluminescence (μPL) measurements as a function of excitation power (shown partially in Figure 9b) on the basis of which the dependence of single emission line intensities was determined (Figure 10a).

## 1. Polarization-resolved photoluminescence

For all the structures characterized by the emission shown in Figure 2, measurements of the polarization-resolved PL were performed. Figure S1 shows the experimental data for all the structures. They served as a base of the degree of linear polarization analysis shown in Figure 4.

**Citation:** Wyborski, P.; Podemski, P.; Wroński, P.A.; Jabeen, F.; Höfling, S.; Sęk, G. Electronic and Optical Properties of InAs QDs Grown by MBE on InGaAs Metamorphic Buffer. *Materials* **2022**, *15*, 1071. https://doi.org/10.3390/ma15031071

Academic Editor: Heesun Yang

Received: 20 November 2021
Accepted: 26 January 2022
Published: 29 January 2022

**Publisher's Note:** MDPI stays neutral with regard to jurisdictional claims in published maps and institutional affiliations.

**Copyright:** © 2022 by the authors. Licensee MDPI, Basel, Switzerland. This article is an open access article distributed under the terms and conditions of the Creative Commons Attribution (CC BY) license (https://creativecommons.org/licenses/by/4.0/).

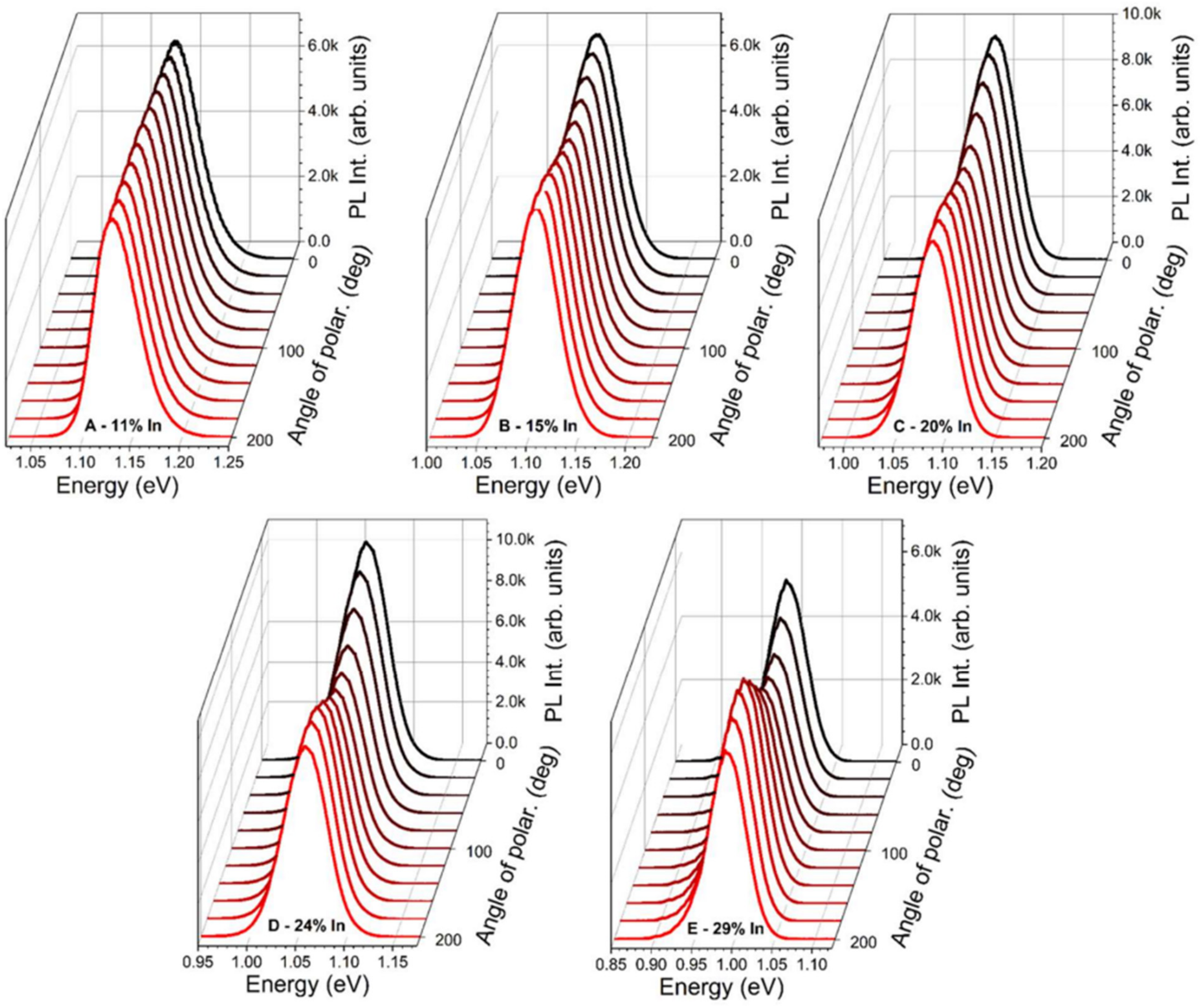


**Figure S1.** PL spectra for structures A-E in the function of linear polarization angle (the scale of polarization angles in the experimental setup coordination system). .

The obtained spectra were integrated in order to determine the PL intensity as a function of the linear polarization angle and then fitted using Formula (1), which allowed determining the degree of linear polarization.

## 2. Temperature-dependent photoluminescence

In this section, we present the results of photoluminescence measurements as a function of temperature for all the structures on the basis of which the influence of the sample temperature changes on the emission could be analyzed in particular, the thermal carrier activation energies and hence the related carrier loss mechanisms in these quantum dots could be determined. Figure S2 shows the temperature-dependent photoluminescence measurements for all the investigated structures.

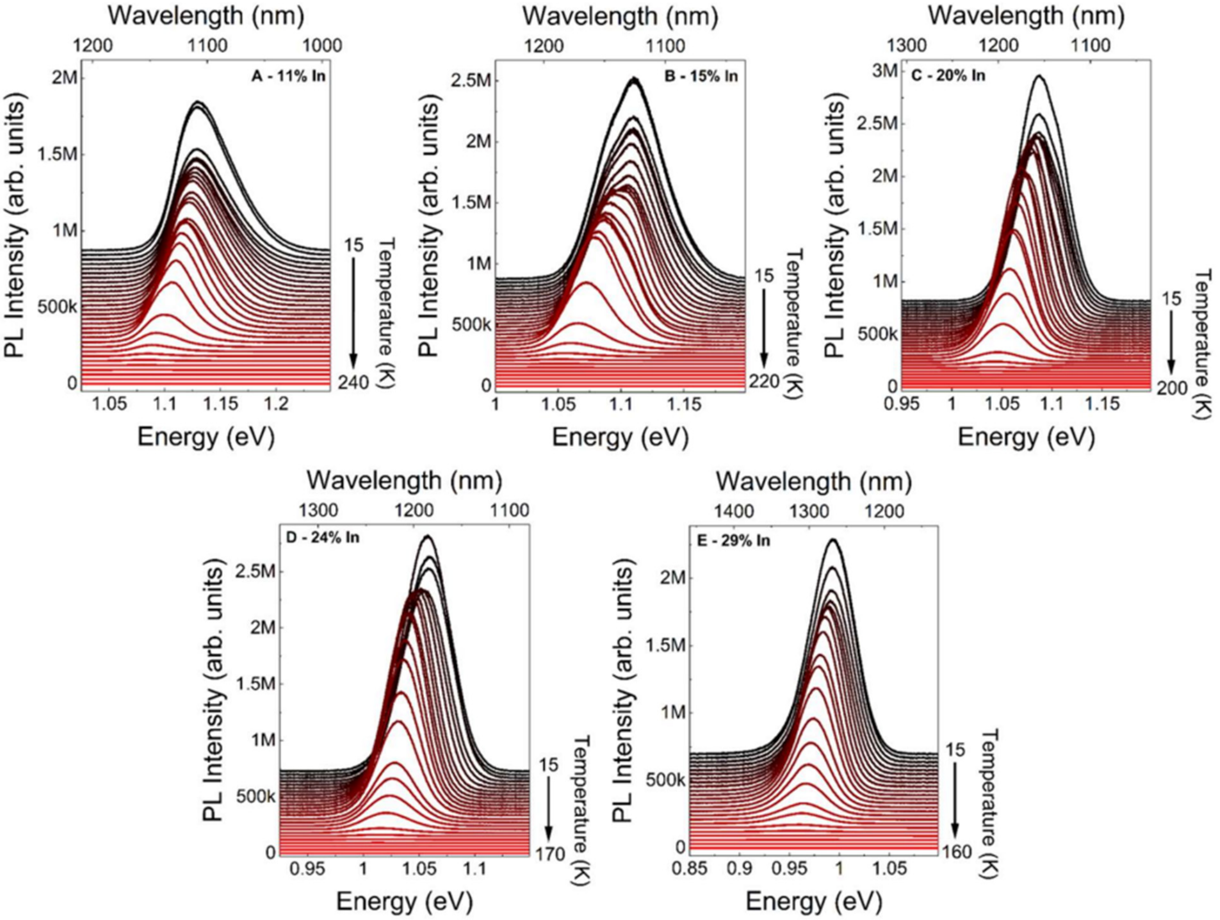


**Figure S2.** PL spectra for structures A-E in a function of temperature.

These spectra were next fitted with Gaussian equation to establish the emission peak energy and full width at half maximum (FWHM), and integrated to determine the intensity of the PL vs temperature for the ensemble of QDs. The results of this analysis are presented in Figures 5 and 6.

## 3. Power-dependent microphotoluminescence

In order to characterize the emission from single QDs, μPL excitation power dependence measurements were carried out. Figure S3 presents the full results of measurements for the selected mesa structure, which are selectively presented in Figure 9b. On the basis of these data, the dependence of μPL intensity was determined versus the excitation power, which are presented in Figure 10a for selected lines marked as X and XX.

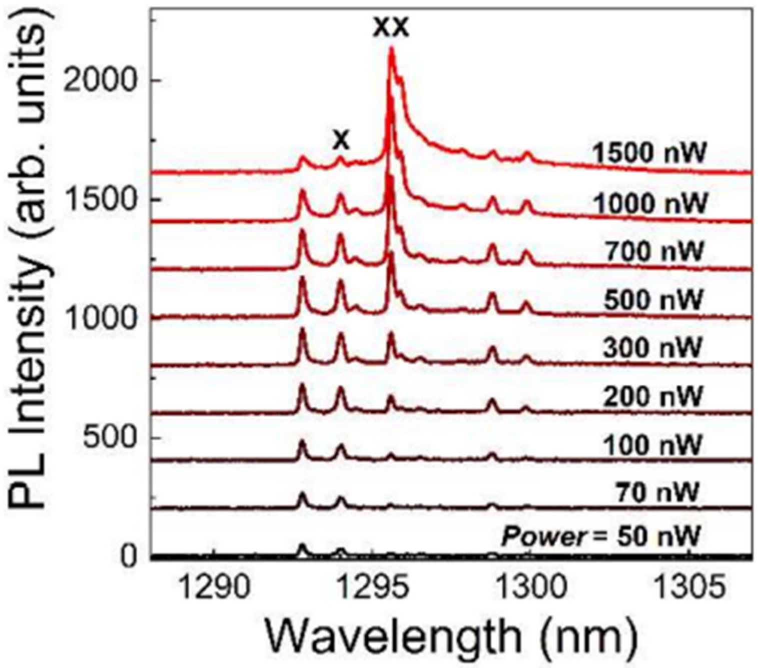


**Figure S3.** μPL spectra for a selected mesa in a function of the excitation power.

The obtained spectra were integrated for particular lines to determine μPL intensity and then fitted using the three-level rate-equation model (ref. 72 in manuscript). The results of this analysis are presented in Figure 10a.
